# Administration of angiotensin II and a bradykinin B2 receptor blocker in midpregnancy impairs gestational outcome in guinea pigs

**DOI:** 10.1186/1477-7827-12-49

**Published:** 2014-06-04

**Authors:** Gloria Valdés, Daniela Schneider, Jenny Corthorn, Rita Ortíz, Stephanie Acuña, Oslando Padilla

**Affiliations:** 1 Centro de Investigaciones Médicas, Escuela de Medicina, Pontificia Universidad Católica, Santiago, Chile; 2 Departamento de Nefrología, Escuela de Medicina, Pontificia Universidad Católica, Santiago, Chile; 3 Departamento de Salud Pública, Escuela de Medicina, Pontificia Universidad Católica, Santiago, Chile

**Keywords:** Renin-angiotensin system, Kallikrein-kinin system, Pregnant guinea-pig, Gestational systemic blood pressure regulation, Utero-placental units, Pregnancy outcomes

## Abstract

**Background:**

The opposing renin-angiotensin system (RAS) and kallikrein-kinin system (KKS) are upregulated in pregnancy and localize in the utero-placental unit. To test their participation as counter-regulators, circulating angiotensin II (AII) was exogenously elevated and the bradykinin B2 receptor (B2R) was antagonized in pregnant guinea-pigs. We hypothesized that disrupting the RAS/KKS balance during the period of maximal trophoblast invasion and placental development would provoke increased blood pressure, defective trophoblast invasion and a preeclampsia-like syndrome.

**Methods:**

Pregnant guinea-pigs received subcutaneous infusions of AII (200 μg/kg/day), the B2R antagonist Bradyzide (BDZ; 62.5 microg/kg/day), or both (AII + BDZ) from gestational day 20 to 34. Non-pregnant cycling animals were included in a control group (C NP) or received AII + BDZ (AII + BDZ NP) during 14 days. Systolic blood pressure was determined during cycle in C NP, and on the last day of infusion, and 6 and 26 days thereafter in the remaining groups. Twenty six days after the infusions blood and urine were extracted, fetuses, placentas and kidneys were weighed, and trophoblast invasion of spiral arteries was defined in the utero-placental units by immunocytochemistry.

**Results:**

Systolic blood pressure transiently rose in a subgroup of the pregnant females while receiving AII + BDZ infusion, but not in AII + BDZ NP. Plasma creatinine was higher in AII- and BDZ-treated dams, but no proteinuria or hyperuricemia were observed. Kidney weight increased in AII + BDZ-treated pregnant and non-pregnant females. Aborted and dead fetuses were increased in dams that received AII and AII + BDZ. The fetal/placental weight ratio was reduced in litters of AII + BDZ-treated mothers. All groups that received interventions during pregnancy showed reduced replacement of endothelial cells by extravillous trophoblasts in lateral and myometrial spiral arteries.

**Conclusions:**

The acute effects on fetal viability, and the persistently impaired renal/placental sufficiency and incomplete arterial remodeling implicate the RAS and KKS in the adaptations in pregnancy. The results partially confirm our hypothesis, as a preeclampsia-like syndrome was not induced. We demonstrate the feasibility of characterizing systemic and local modifications in pregnant guinea-pig, supporting its use to study normal placentation and related disorders.

## Background

The renin-angiotensin system (RAS) has traditionally been considered the main vasopressor stimulus. This was because angiotensin II (AII), which was considered the terminal peptide of the RAS, exerts a potent effect when binding to its AT1 receptor (AT1R). However, the functional scope of the RAS and the number of its participants have now been extended [1] by non-proteolytic RAS activation through the renin/prorenin receptor [2], a vasodilating receptor of AII (AT2R) and several intermediate vasodilatory peptides/receptors, including angiotensin-(1–7)/Mas, angiotensin-(3–8) or AIV/AT4R, and angiotensin-(1–9)/AT2R [3-7].

The kallikrein-kinin system (KKS) includes both a plasma and a tissue cascade. Plasma kallikrein is synthesized in the liver, released into circulation, and activated by coagulation factor XII to liberate bradykinin and lys-bradykinin from high-molecular-weight kininogen. The main tissue kallikrein, KLK1, is synthetized in various organs and releases lys-bradykinin by cleaving low-molecular-weight kininogen. Kinins activate the main constitutive B2 receptor (B2R) and the B1 receptor, the latter of which is scarcely expressed in normal tissues and is mainly induced by tissue injury, endotoxins, or cytokines [8]. The tissue KKS has a vasodepressor role, which has been unmasked by converting enzyme inhibitors [9-11] and blockers of its main vasodilatory constitutive receptor, the bradykinin B2R [12].

The enzymatic cascades of the RAS and the KKS are virtually mirror images of each other. Bradykinin and angiotensin II enhance angiogenesis while angiotensinogen is antiangiogenic [13], and the angiotensin II/AT1R and bradykinin/B2R pathways are proinflammatory. However, both peptidergic systems have antagonic vasomotor and pleiotropic effects, impacting vascular smooth muscle, fibrosis, cardiovascular hypertrophy, platelet aggregation, oxidative stress and electrolyte balance. The two systems are ubiquitous and have endocrine, paracrine, and autocrine roles [14,15]. Moreover, they maintain active cross-talk at various sites, from activation of prorenin by kallikrein [16,17] to heterodimerization of their main receptors AT1R and B2R [18-21]. Overall, their vasodilating peptides integrate a rich network of potentiating pathways [22].

During gestation, the RAS and the KKS are associated with up- or down-regulation of maternal blood pressure, and with auto/paracrine effects at the feto-maternal interface [19,23-26]. Moreover, in vitro, the invasive capacity of immortalized trophoblasts is impaired by angiotensin II [27] and enhanced by bradykinin B2R-mediated stimulation [28]. Thus, we believe that the RAS and KKS are interrelated antagonistic systems that should be jointly studied.

The guinea-pig is an attractive model for such studies, as it shares with humans an extensive vascular transformation [29], a hemomonochorial placenta, the temporal pattern of progesterone levels [30], a preeclamptic-like syndrome associated with reduced placental perfusion [31], a utero-placental repertoire of vasoactive and angiogenic factors in functionally equivalent structures [22,32-35] which includes the B1 and B2 [36] as well as the AT1 and AT2 bradykinin and angiotensin receptors respectively (Acuña and Valdés, unpublished observation).

Here we tested our hypothesis that disturbing the endogenous balance between the RAS and the KKS in pregnant guinea-pigs, during the period of maximal trophoblast invasion and placental development [37], by the administration of angiotensin II or Bradyzide – a potent non-peptide antagonist of the B2R [38] - would provoke a defective trophoblast invasion, fetal losses, blood pressure changes and preeclampsia-like morphological and functional alterations.

## Methods

### Animals

All experiments were conducted according to the Guide for the Care and Use of Laboratory Animals (National Research Council, USA), and were approved by the Institutional Review Board for Ethics and Animal Welfare, and by the Ethics Committee of FONDECYT (Fondo Nacional de Desarrollo Científico y Tecnológico, Chile).

Virgin Pirbright white guinea-pigs of ~600 g were kept under controlled conditions of humidity and temperature (25°C), with a 12-h light–dark cycle. Females were examined daily for perforation of the vaginal closure membrane. Upon this occurrence, they were caged with fertile males, and the following day was defined as day 1. Between days 18–20, pregnancy was confirmed by ecography (Aloka Flexus SSD-1100; Hitachi Aloka Medical, Tokyo, Japan). On day 20, pregnant guinea-pigs were anesthetized with intraperitoneal ketamine (60 mg/kg) and xylazine (4 mg/kg) prior to subcutaneous implantation of Alzet 2ML2 osmotic pumps (Durect, Cupertino, CA) in the interscapular region.

Over 14 days, these pumps delivered either saline solution (control; n = 6), angiotensin II (Sigma) 200 μg/kg/day (AII; n = 7), Bradyzide (Sigma) 62.5 μg/kg/day (BDZ; n = 5), or angiotensin II plus Bradyzide (AII + BDZ; n = 6). The dose of AII was subpressor, as determined in a pilot study in non-pregnant guinea-pigs. To evaluate the direct and the persistent consequences of the interventions, systolic blood pressure was determined during the last hours of the infusion on gestational day 34, and at 6 and 26 days after withdrawal of the osmotic pumps. Blood pressure was measured under anesthesia in the pad of the right paw compressed by a neonatal blood pressure cuff (Critikon, General Electric Healthcare, Connecticut, EEUU) using a Power Lab 8 SP system (ADInstruments, Sidney, Australia). Blood pressure data were analyzed with the Labchart graphic software 6.1 Pro (ADInstruments). After the blood pressure measurement on gestational day 34, the osmotic pumps were extracted while the animals were still anesthetized.

The study also included two groups of non-pregnant cycling guinea-pigs. The first group included control non-pregnant females (C NP; n = 5); in them the systolic blood pressure was measured in random days of the estrous cycle. The second group received subcutaneous infusions of AII + BDZ for 14 days (AII + BDZ NP; n = 6), and systolic blood pressure was measured on the last day of infusion, and at 6 and 26 days after its discontinuation.

On gestational day 60 or 26 days after AII + BDZ infusion in non-pregnant females, with the animals under ketamine and xylazine, urine was withdrawn from the bladder for determination of protein (Bradford method) and creatinine (Beckman Autoanalyser, Fullerton, CA) and blood from the left ventricle for creatinine and uric acid determination (Beckman Autoanalyser). Then the animals were euthanized with an overdose of ketamine/xylazine, and the uterus, feto-placental units, and kidneys were removed. The fetuses, placentas, and kidneys were weighed after trimming off the umbilical cord, amniotic membranes, and perirenal fat. Fetal loss was attributed to demise if an atrophic or necrotic feto-placental unit was observed, or to abortion when a fetus observed by ultrasonography was absent at term, since miscarriages in guinea-pigs are frequently not accompanied by persistence of the implantation site (Elger, personal communication). Only units with a live fetus were included for measurements of fetal and placental weight and for immunohistochemical studies. A central cross-section through the placenta, subplacenta, implantation site, and underlying myometrium was fixed as a single block. The placenta, and the subplacental decidua and myometrium were also isolated in other feto-placental units; from these, the fetal/placental weight ratio was calculated as a marker of placental efficiency [39]. Tissues were immediately fixed with phosphate-buffered 10% formalin for 24 h, then dehydrated in a graded series of ethanol and xylene dilutions, and embedded in Paraplast-Plus® (Sigma, St. Louis, MO). Sections (6 μm) were mounted on silanized slides.

### Immunostaining procedure

Immunostaining was performed at room temperature. Deparaffinized sections were rehydrated using ethanol, rinsed three times for five minutes each in phosphate-buffered saline with 50 mM Tris–HCl, and submitted to heat-induced antigen retrieval using citrate buffer (pH 6.0). Endogenous peroxidases were blocked by incubation in 10% H_2_O_2_ for ten minutes. Sections were then incubated in a humid chamber for 30 min with protein block (Cas-Block®; Zymed, San Francisco, CA), followed by incubation for 18 h at 4°C with anti-pancytokeratin mouse monoclonal antibody (1:50, P2871, Sigma). Sections were immunostained using a biotin-streptavidin-peroxidase system (LSAB + ®, DakoCytomation). Finally, the samples were treated for 15 min with 0.1% (w/v) 3-3′-diaminobenzidine in buffer containing 0.05% H_2_O_2_. The slides were counterstained with Harris hematoxylin (Sigma).

### Evaluation of uterine arterial trophoblast invasion

Spiral arteries were identified as lateral if localized in the periphery of the subplacenta, or myometrial if in the uterine smooth muscle below the placental bed. The extravillous trophoblast (EVT) was defined as intramural when located in the media of the spiral artery, and as endovascular when lining the lumen of the spiral artery and replacing endothelial cells. Photographic images were acquired with a Nikon CoolPix 4500 camera (Nikon Inc., Tokyo, Japan), coupled to a Zeiss AxioImager AX.10 microscope (Carl Zeiss, CA). The portion of the endovascular trophoblast replacing endothelial cells was calculated by measuring the arterial perimeter occupied by cytokeratin-positive cells, using Axiovision 4.8.2.0 LE (Carl Zeiss AG, Inc., Oberkochen, Germany). Groups were compared according to the percentage of intraluminal perimeter occupied by cytokeratin-positive cells in the lateral and myometrial spiral arteries.

### Statistics

A Bayesian analysis [40] was performed to test differences between systolic blood pressures in different groups and periods and the proportion between viable versus lost fetuses according to treatment. Systolic pressure was modeled as a multivariate normal distribution with different mean vectors and variance-covariance matrices for groups and periods. Data are expressed as mean and dot-plot of individual values. The logarithm of the non-viable fetuses/[1 – the predicted non-viable fetuses] was modeled as a normal distribution, with group-dependent median and variances. After proving that variances were similar among groups, a common variance was used. For each group, we calculated the proportion of viable fetuses, the differences between these proportions, and their credible intervals (CI). Data are expressed as proportion of live fetuses and CI. The analyses were performed with the open-source OpenBUGS program. Differences were considered significant when the CI did not cross zero.

A one-way ANOVA with Fisher’s LSD post-hoc test was performed to determine the effects of the interventions on fetal, placenta, and kidney weights. Student’s t-test was used to evaluate differences in the protein/creatinine index among the different groups of pregnant dams and the non-pregnant females that received AII + BDZ. Data are expressed as mean and dot-plot of individual values. Data were analyzed using Graphpad Prism 6.01 (GraphPad Inc., San Diego, CA) and were considered significantly different when P < 0.05.

## Results

### Maternal blood pressure

**Figure 1:**
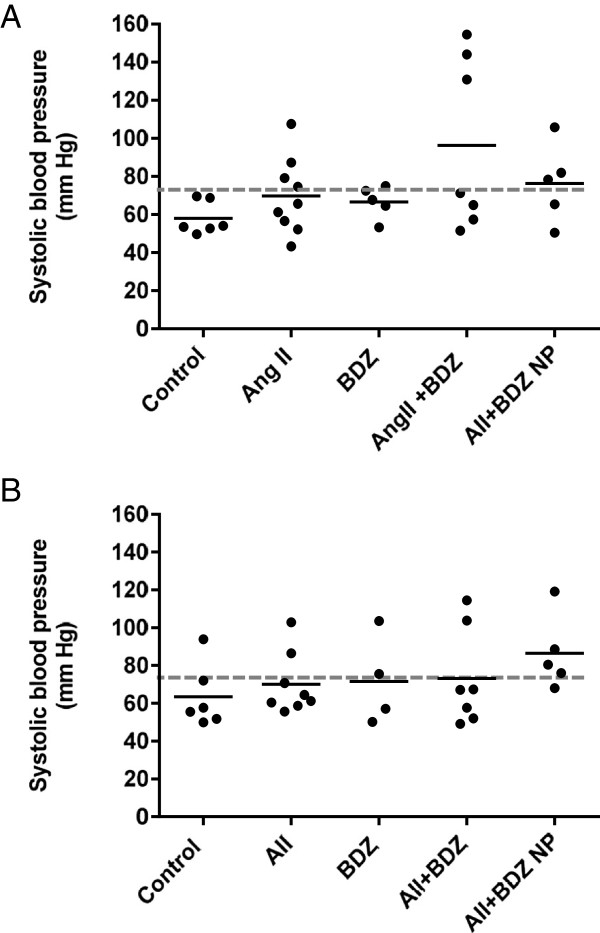
**Systolic blood pressure in pregnant and non-pregnant guinea-pigs treated with angiotensin II (AII), bradyzide (BDZ), or AII + BDZ.** Systolic blood pressure on gestational days 34 **(A)** and 60 **(B)** in controls (C), AII, BDZ and AII + BDZ treated dams and in AII + BDZ-treated non-pregnant females (AII + BDZ NP). The broken line depicts the mean systolic blood pressure of 73.2 ± 7.5 SEM mm Hg in untreated non-pregnant females (C NP); continuous line represents mean values.

Pregnant dams in gestational day 34, which were on the fourteenth day of AII + BDZ infusion, exhibited a dual effect; in them a group displayed a marked increase of systolic blood pressure while another maintained values within that of control animals and those receiving single infusions (Figure 1A). This elevation subsided after infusion cessation, such that the systolic blood pressure at gestational day 40 (not shown) and 60 were similar to those in the control and singly treated groups (Figure 1B). The blood pressure elevation induced by AII + BDZ in a subgroup of pregnant animals did not occur in AII + BDZ-treated non-pregnant females. (Figure 1A).

### Fetal, placental, and maternal outcomes

**Figure 2:**
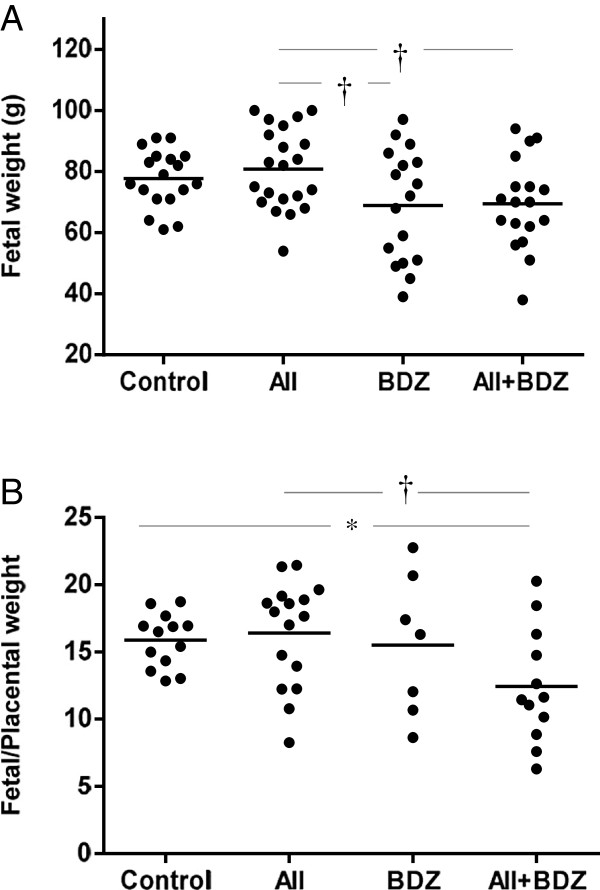
**Fetal weight and fetal/placental weight ratios from dams treated with saline, angiotensin II (AII), Bradyzide (BDZ), or AII + BDZ. (A)** Fetal weight. **(B)** Fetal/placental weight ratio. Continuous line represents mean values. *P < 0.05; †P < 0.01.

The proportions of viable versus lost fetuses (including abortions and fetal demises) were reduced in the groups treated with AII (0.75; CI, 0.57–0.89) and with AII + BDZ (0.68; CI, 0.50–0.84) compared to the control group (0.95; CI, 0.8–0.99, P < 0.05); the ratio of viable/lost fetuses did not differ between the control and BDZ-treated groups (0.80; CI, 0.57–0.95). Infusions with BDZ and AII + BDZ were associated with reduced fetal weight, compared to animals infused with only AII (P < 0.01) (Figure 2A). The fetal/placental weight ratio was also reduced in litters of mothers treated with AII + BDZ, compared to those of controls (P < 0.05) and the AII group (P < 0.01) (Figure 2B). The placental weights of the treated dams were not different from those of controls.

Kidney weights were similar in all groups. AII-treated dams had higher plasma creatinine levels than controls (0.46 ± 0.05 versus 0.33 ± 0.02 mg/dl; P < 0.01). Both pregnant and non-pregnant animals receiving AII + BDZ presented reduced plasma creatinine levels compared with animals receiving single interventions (0.38 ± 0.02 and 0.36 ± 0.02 mg/dl in comparison to 0.46 ± 0.05 and 0.48 ± 0.04 mg/dl for AII and BDZ; P < 0.05). Neither control, treated dams or non-pregnant AII + BDZ treated females exhibited proteinuria or hyperuricemia.

### Remodeling of uterine arteries

**Figure 3:**
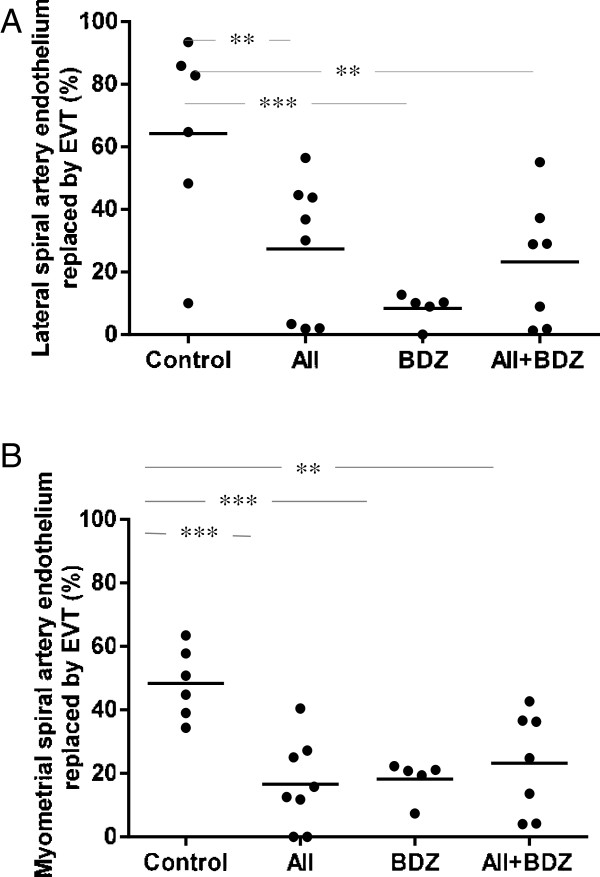
**Intraluminal extravillous trophoblasts in spiral arteries on gestational day 60 in dams treated with saline, angiotensin II (AII), Bradyzide (BDZ), or AII + BDZ. (A)** Lateral spiral artery endothelium replaced by extravillous trophoblasts (EVT) (% perimeter) in dams treated with saline, AII, BDZ, or AII + BDZ. **(B)** Myometrial spiral artery endothelium replaced by EVT (% perimeter) in dams treated with saline, AII, BDZ, or AII + BDZ. Continuous line represents mean values. **P < 0.005; ***P < 0.0005.

**Figure 4:**
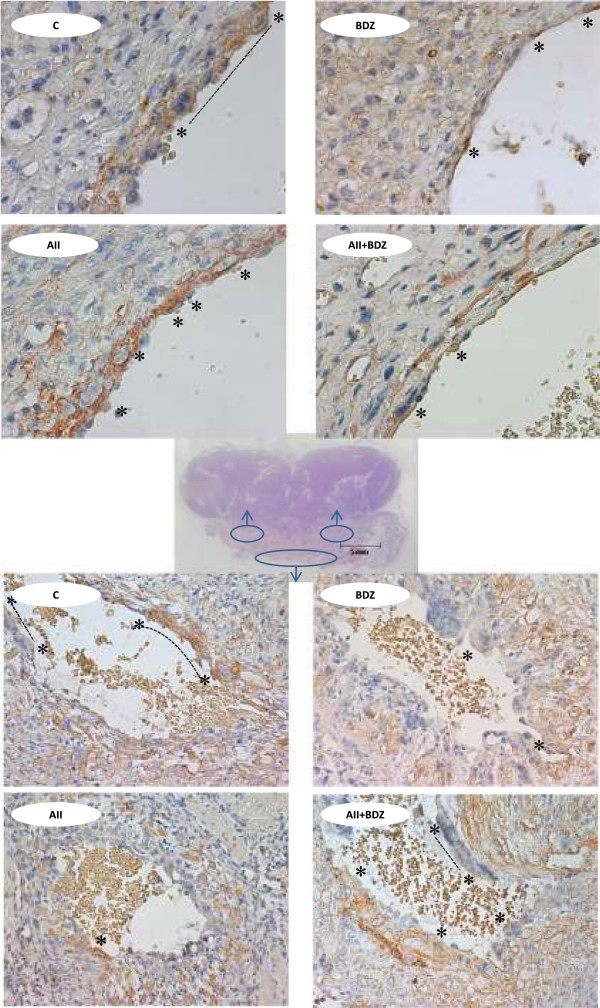
**Representative sections of spiral arteries on day 60 of gestation in dams treated with saline (C), Bradyzide (BDZ), angiotensin II (AII) angiotensin II + Bradyzide (AII + BDZ).** Endoluminal trophoblasts in lateral and myometrial spiral arteries are depicted in the upper and lower panel, respectively. The whole-mount haematoxylin/eosin section of the guinea-pig utero-placental interface includes in blue ovals the zones from which the microphotographs were acquired; trophoblasts were identified by anti-cytokeratin and highlighted by * and interrupted lines. Magnification x400 in upper panel and x100 in lower panel.

In the lateral spiral arteries no intramural trophoblasts were observed; all treated groups exhibited decreased intraluminal replacement of endothelium by extravillous trophoblasts (P < 0.005 for the AII-, AII + BDZ- and P < 0.0005 for the BDZ-treated groups versus control) (Figure 3A). In myometrial spiral arteries, the perimeter occupied by endoluminal EVT was reduced in the groups receiving AII, BDZ, (P < 0.0005 for both) and AII + BDZ (P < 0.005) as compared to controls. (Figure 3B). In summary, both the isolated and combined interventions resulted in reductions of endoluminal EVT in lateral and myometrial spiral arteries as depicted in Figure 4, upper and lower panel, respectively.

## Discussion

The results of the present study indicate that a transient disruption of the balance between the RAS and the KKS in favor of the RAS in midpregnancy alters the maternal adaptation to pregnancy.

At the level of the utero-placental unit, tilting the RAS/KKS balance towards vasoconstriction caused fetal losses and placental insufficiency, and reduced the replacement of endothelium by endovascular trophoblasts in the spiral arteries. Infusions were delivered between gestational days 20 and 34, which is when extravillous trophoblasts display the maximal decidual penetration [37]; thus the secondary fetal losses may be attributed to decreased decidual vasodilatation, hyperpermeability, and priming of the spiral arteries [41]. The retardation of trophoblast invasion, reflected at term by a reduction of endovascular trophoblasts in viable feto-placental units, was likely due to B2R blockade and excessive AT1R stimulation as observed in HTR-8/SVneo cells [27,28]. Guinea-pigs express AT1 and AT2 receptors in the placenta, decidua, and extravillous trophoblasts (Acuña and Valdés, unpublished observation), similar to what has been described in humans [26,32,42-45]. However, the deleterious effects of AT1R antagonists in pregnancy [46,47] preclude their use for identifying the main receptor stimulated by excess angiotensin.

The acute effect of angiotensin II plus a B2R blocker provoked an increase of systolic blood pressure in a subset of the group (responders) to values that were not attained with single interventions and in non-pregnant females receiving the combined treatment. The responders are probably bradykinin-dependent when circulating angiotensin II surpasses the endogenous gestational levels.

This study leaves unresolved questions regarding why additive effects of combined interventions were observed for some, but not all, study parameters. The discordance between the lack of additive effect on fetal weight and the summatory impact on the fetal/placental weight ratio could be because the combined intervention induces greater fetal losses, which favor the growth of the remaining units. The higher plasma creatinine in dams that received BDZ—absent in the AII + BDZ group—could be attributed to a lack of the protective effect of B2R in the face of gestational hyperfiltration [48], as reported in B2R-null mice [49]. In the AII + BDZ group, this could be compensated by positive regulation of *BdkrB2* gene expression by AII, and by AT1R-mediated activation of B2R expression [50]. These postulates exemplify how, *in vivo*, an intricate vasodilatory/pleiotropic network and tissue-specific effects could diminish the effects of angiotensin II stimulation and B2R blockade.

The presently observed persistent changes in trophoblast invasion partially confirm our hypothesis that disturbing the endogenous balance between the RAS and the KKS in mid-pregnancy would provoke a defective trophoblast invasion. However, the preeclampsia phenotype was not reproduced. It remains to be tested whether dams would develop this syndrome if the interventions were continued to term. In humans, a maintained increase in circulating AII could correlate with the agonistic autoantibody to AT1R [23], the T235 polymorphism of the angiotensinogen gene [51], and the redox conformation of angiotensinogen that facilitates angiotensin release [24]. Decreased stimulation of the B2R by bradykinin could derive from a depressed KKS, as observed in women who present with pregnancy hypertension or preeclampsia [52,53]. Lastly, the AII + BDZ combination might resemble the heterodimerization of the AT1R and B2R, which sensitizes the AT1R and blunts the response of the B2R [19]. A persistent disequilibrium of the vasoconstrictor/vasodilator balance would be magnified along its course by the recruitment of intermediate effectors.

## Conclusions

By transiently tilting the balance of the opposing RAS and KKS systems during the period of maximal trophoblast invasion, we demonstrated deleterious effects of AII and B2R blockade in pregnant guinea-pigs. Further understanding of the effects of the opposing RAS and KKS could inspire the development of pharmacological interventions to enhance the KKS in order to counteract excessive preponderance of AT1R activation in maternal circulation and the utero-placental interface in preeclampsia, mediated by angiotensin II and the agonistic AT1R autoantibody. This study also demonstrates the feasibility of characterizing systemic and local modifications in the pregnant guinea-pig, supporting the use of this model in studies of normal placentation and related disorders.

## Competing interests

The authors declare that they have no competing interests.

## Authors’ contributions

GV designed the study, participated in sample extraction and data analysis, drafted the manuscript and wrote its final version. DS and JC implanted the osmotic pumps, and performed blood pressure measurements and animal dissections. DS performed the immunohistochemistry, the digital processing of the images, and the statistical analysis. RO performed the ultrasonographies and Stephanie Acuña the acquisition of the microphotograhs. Oslando Padilla supervised the statistical analysis and performed the Bayesian statistics. With the exception of deceased JC, all authors read and approved the final manuscript.
